# Multi-Material Implants for Temporomandibular Joint Disc Repair: Tailored Additive Manufacturing Production

**DOI:** 10.3389/fbioe.2020.00342

**Published:** 2020-04-21

**Authors:** Carla Moura, Daniela Trindade, Milena Vieira, Luís Francisco, David Faustino Ângelo, Nuno Alves

**Affiliations:** ^1^Centre for Rapid and Sustainable Product Development, Polytechnic Institute of Leiria, Leiria, Portugal; ^2^ESTG – School of Technology and Management, Polytechnic Institute of Leiria, Leiria, Portugal; ^3^SEG-CEMMPRE – Department of Mechanical Engineering, University of Coimbra, Coimbra, Portugal; ^4^Faculdade de Medicina da Universidade de Lisboa, Lisbon, Portugal; ^5^Instituto Português da Face, Lisbon, Portugal

**Keywords:** temporomandibular joint disc, processing conditions, Poly(ε-caprolactone), Poly(ethylene glycol) diacrylate, multi-material structures

## Abstract

Temporomandibular disorders (TMD) affect a substantial percentage of the population, and the resources spent on their treatment are considerable. Despite the worldwide efforts around Tissue Engineering of the temporomandibular joint (TMJ) disc, a proper implant offering a long-term solution for TMD was not yet developed. To contribute to these efforts, this work is focused on the research and development of implants for TMJ disc regeneration. Scaffolds and hydrogels mimicking the TMJ disc of black Merino sheep were produced using different materials, poly(ε-caprolactone) (PCL) and poly(ethylene glycol) diacrylate (PEGDA), and as a multi-material structure. Different parameters of the scaffold manufacturing were assessed: the influence of processing temperatures, filament diameter, and biological environment. Moreover, two multi-material approaches were also assessed, scaffold with a hydrogel shell and scaffold with a hydrogel core. It was found that increasing temperature, the scaffolds’ porosity decreases, increasing their compressive modulus. Decreasing the filament size (300 to 200 μm) decreases the compressive modulus to almost half of the initial value. Scaffolds with 200 μm filaments are the ones with a closer modulus to the native disc and their properties are maintained under hydrated conditions. The introduction of a hydrogel core in these scaffolds presented better mechanical properties to TMJ disc substitution.

## Introduction

The temporomandibular joint (TMJ) is the articulation between the glenoid fossa and the articular eminence of the temporal bone and the mandibular condyle, with an interposed fibrocartilaginous disc. Problems in the TMJ are the most common cause of chronic or recurrent orofacial pain (Yap and Toh, 2016).

Temporomandibular disorders (TMD) are a heterogeneous class of pathologies associated with the masticatory musculature, TMJ and surrounding bony and soft tissues structures (Liu and Steinkeler, 2013; Yap and Toh, 2016). It is estimated that 41% of the world population experiences TMD symptoms throughout their lifetime (Okeson, 2014) and that only 3–7% seek treatment (Murphy et al., 2013). Several studies indicate that TMD symptoms are more common in females and in the young and middle-aged adult population (Manfredini et al., 2011; KÖhler et al., 2012; Minghelli et al., 2014b). For patients with TMD, the prevalence is estimated to be 45% for muscle disorders, 41% for disc displacement and 30% for other joint disorders (Manfredini et al., 2011). Moreover, TMD have been related to other disturbances, such as anxiety and depression (Athanasiou et al., 2009; Willard et al., 2011; Minghelli et al., 2014a). In Portugal, studies indicate that TMD symptoms are experienced by 42% of the adult population (31% in males and 48% in females) (Minghelli et al., 2014b) and 25% of the young population (Minghelli et al., 2014a).

Temporomandibular disorders symptoms include pain in the joint and surrounding muscles, clicks, discomfort when moving the jaw, teeth grinding (bruxism), headaches and reduced jaw motion or locking of the jaw. Internal derangement and degenerative joint disease (osteoarthritis, OA) are conditions that usually end up requiring surgical treatment (Willard et al., 2011).

Temporomandibular disorders clinical treatments are usually divided into four categories, (i) non-invasive, (ii) minimally invasive, (iii) invasive, and (iv) alloplastic replacement (Tanaka et al., 2008; Athanasiou et al., 2009; Murphy et al., 2013). However, to date, there is no permanent treatment for TMD. Tissue Engineering (TE) may offer a permanent solution to eliminate symptoms of these disorders and restore joint function. TE is a multidisciplinary field which combines the principles of life sciences (cells and suitable factors, either biochemical, such as growth factors, or physical, such as cyclic mechanical loading) and engineering technologies to provide biological substitutes to functionally repair, regenerate, or replace injured tissues and organs and advanced surgical techniques (Berthiaume et al., 2011; Ratner et al., 2013; Morouço et al., 2016).

Scaffolds are interconnected porous networks which promote the necessary interactions for the formation and regeneration of new functional tissues (Ratner et al., 2013). Hydrogels are three-dimensional (3D) water-swollen networks of crosslinked polymers that have been widely used in biomedical applications, from controlled drug delivery systems to TE (Zhu and Marchant, 2011; El-Sherbiny and Yacoub, 2013). These substitutes, hydrogels and/or scaffolds, should respond to several biological requirements (allow cellular interaction, adhesion, proliferation, migration, and/or differentiation) and mechanical requirements (mimicking the morphological structure, as well as its function) (Morouço et al., 2016).

In biofabrication one can obtain the desired constructs through several different techniques, but most of the procedures presented in this work involved the use of fused deposition modeling (FDM). This additive manufacturing (AM) technology consists on the extrusion of thermoplastic or wax (usually supplied as filament or pellets) through a computer-controlled deposition nozzle that draws the desired feature layer-by-layer (Domingos et al., 2012; Vaezi et al., 2012).

Tissue Engineering of the TMJ disc is a relatively recent field. The first study on TMJ disc TE *in vitro* was published by Thomas et al. (1991). TMJ disc TE attempts to respond to the lack of regeneration and self-repairing capacity of this fibrocartilaginous tissue (Willard et al., 2011). Fibrocartilage is mostly composed of type I collagen fibers, presents low glycosaminoglycans (GAG) content and higher mechanical properties when compared to hyaline cartilage (Johns and Athanasiou, 2007; Athanasiou et al., 2009; Willard et al., 2012; Fermor et al., 2015). Moreover, while hyaline and elastic cartilage are rich in chondrocytes and chondroblasts, fibrocartilage present a cell population of fibroblast and chondrocytes (Detamore et al., 2006; Mescher, 2016).

Several materials have been used to produce artificial ECM supports for TMJ disc regeneration. Frequently used materials include polyamide, polyglycolic acid (PGA), poly(glycerol sebacate) (PGS), polylactic acid (PLA), poly-L-lactic acid (PLLA), polytetrafluoroethylene (PTFE), and other natural biomaterials, such as collagen hydrogels or decellularized pig urinary bladder (Springer et al., 2001; Allen and Athanasiou, 2008; Brown et al., 2012; Hagandora et al., 2013; Juran et al., 2015; Kobayashi et al., 2015). Over the last years, poly(ε-caprolactone) (PCL) has been widely investigated, due to its slow degradation rate, for producing scaffolds, electrospun fibers or composites for cartilage TE, considering the slow rate of cartilage regeneration (Annabi et al., 2011; Garrigues et al., 2014; Legemate et al., 2016; Olubamiji et al., 2016). In addition to having excellent biocompatibility and adequate mechanical properties, it has been widely used for the production of complex structures by AM for craniofacial defects reconstruction (Aldaadaa et al., 2018).

Poly(ethylene glycol) diacrylate hydrogels are also widely studied for cell encapsulation in order to repair cartilage damages in patients with OA (Musumeci et al., 2011a, b, 2012). It is also known that PEG hydrogels suffer a slow *in vitro* hydrolytic degradation, in which its increase in concentration diminish its degradation rate, enabling them for a long-term implant (Choi et al., 2019). This degradation happens due to the cleavage of its ester linkage. Browning et al. (2014) reported a significant *in vivo* degradation of PEGDA hydrogels within 12 weeks (Browning et al., 2014).

Despite worldwide efforts around TMJ disc TE, a proper implant, mimicking the TMJ disc properties and biomechanical environment and offering a long-term solution for TMD, has not been developed yet. To contribute to these efforts, this work is focused on the research and development of implants for TMJ disc regeneration through the manipulation of PCL and PEGDA scaffolds, which has not been developed and tested for this purpose.

The aim of the work is to: (i) produce and optimize a 3D artificial ECM using AM technologies, through the use of an in-house developed extrusion equipment which allows the full control over the production of the scaffolds, (ii) combine different materials to obtain hierarchical and multifunctional structures and (iii) characterize the produced structures, both morphologically and mechanically toward TMJ disc substitution and complete regeneration over time. The materials used in the construction of the desired structures are biocompatible and approved by the Food and Drug Administration (FDA) –PCL and poly(ethylene glycol) diacrylate (PEGDA).

## Materials and Methods

### Scaffold Production

Poly(ε-caprolactone) (MW 6500, Perstorp, Malmo, Sweden) scaffolds were produced using the BioExtruder (CDRSP, IPLeiria) and different production parameters were evaluated, namely (i) nozzle (extrusion head) temperature, (ii) fiber diameter and (iii) the influence of hydrated environment. PCL scaffolds geometry was obtained by reverse engineering a Black Merino sheep TMJ disc (Ângelo et al., 2016) and the fiber alignment was 0 and 90°.

To assess the influence of the nozzle temperature on the scaffolds morphology and mechanical behavior, PCL scaffolds were produced using three nozzle temperatures: 78°C (T78), 80°C (T80) and 86°C (T86). A 300 μm nozzle was used, which will correspond to the final fiber diameter. The deposition spindle speed was 10.5 rpm for group T78 and 14.5 rpm for groups T80 and T86, while the crosshead speed was 9 mm⋅s^–1^ for the group T78 and 12 mm⋅s^–1^ for groups T80 and T86.

In the second stage, the influence of fiber diameter on the scaffolds’ morphology and mechanical environment was assessed. PCL scaffolds were produced at 78°C using a nozzle with a diameter of 300 μm (∅300) and 200 μm (∅200). Scaffolds from ∅300 group were produced with the same parameters as T78 group, while the scaffolds from the ∅200 group were produced using a deposition and crosshead velocities of 50 rpm and 5 mm⋅s^–1^.

To evaluate the influence of a hydrated environment, scaffolds with the characteristics of each ∅300 and ∅200 group were submerged in distilled water (dH_2_O), at 37°C for 24 h (BIO ∅300 and BIO ∅200 groups, respectively).

### Hydrogel Production

Poly(ethylene glycol) diacrylate (MW 575, Sigma-Aldrich^®^) hydrogels were produced at 20% (V/V), dissolved in a 0.5 M aqueous solution of 2-[4-(2-hydroxyethyl)piperazin-1-yl]ethanesulfonic acid (HEPES) buffer (Sigma-Aldrich^®^). Photopolymerization was induced through the addition of 0.1% (w/V) 2,2-dimethoxy-1,2-diphenylethanone (DMPA, Sigma-Aldrich^®^) to 10 mL of PEGDA solution in a transparent Petri dish. Before UV light (λ = 365 nm) exposure, the mixture was heated at 45°C for 30 min to integrate the photoinitiator with the blend. Hydrogel formation takes about 3 min to fully exhaust the acetylate groups. The hydrogels’ form was obtained using a cutting tool, whose mold was produced based on the shape of a Black Merino sheep TMJ disc that was previously scanned and digitized.

### Multi-Material Structures

To better replicate the native environment, two multi-material approaches to produce a TMJ disc were investigated using a combination of the two materials previously studied. It was hypothesized that the combination of a scaffold and a hydrogel would result in better implant performance. In this case, PCL scaffolds would provide mechanical strength and (i) a shell or (ii) a core made of PEGDA would lubricate, diminish tension between surfaces and improve the properties of the final construct. In the first approach, a layer of PEGDA was photopolymerized surrounding the PCL scaffold. In the second, the PEGDA hydrogel was injected into the PCL scaffolds filling the pores. In both cases, PCL scaffolds were produced as previously described and submerged in sodium hydroxide (NaOH) for 24 h to change the hydrophobic behavior of the PCL.

### Mechanical Properties

The structures’ mechanical behavior was assessed by uniaxial unconfined compression tests using a universal testing machine with a 1 mm min^–1^ extension rate. Before mechanical testing, the thickness, 10 (mm), of each structure was determined using a digital caliper (Bocchi Control), and the area, A_0_ (mm^2^), was determined using ImageJ2 software. Additionally, prior to mechanical evaluation, PEGDA hydrogels and the multi-material scaffolds with injected PEGDA (core) were placed in dH_2_O for 24 h to ensure the complete hydration of the hydrogel.

Force, F (N), and extension, l (mm), were recorded at any given moment throughout the tests. Compressive stress, σ (MPa), was determined using:

(1) $$\sigma =\text{F}/{\text{A}}_{0}$$

The strain, ε was determined using:

(2) $$\varepsilon =\left(\text{l}-{\text{l}}_{0}\right)/{\text{l}}_{0}$$

The compression modulus (E) was calculated from the slope of the linear region of the stress-strain curve (*r*^2^ > 0.99).

### Micro-Computed Tomography

**FIGURE 1:**
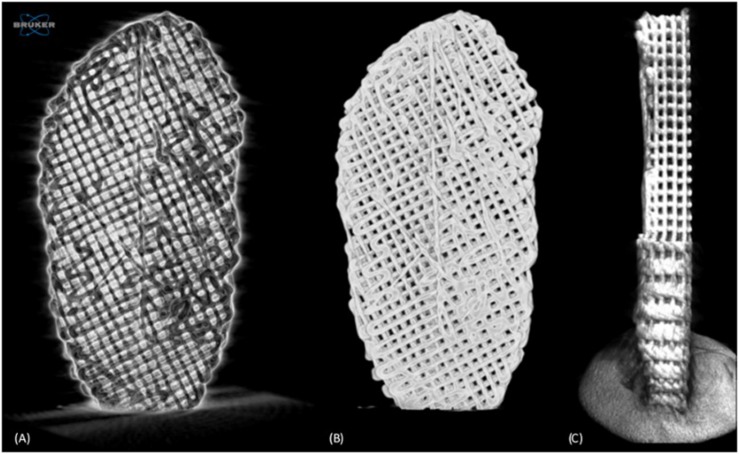
Micro-computed tomography (μCT) analysis. Negative of the scaffold scan with noise **(A)**, scaffold reconstruction after filtering and application of threshold **(B)**, and scaffold sectioned image **(C)**.

A SkyScan 1174^TM^ (software version 1.1, Bruker, Kontich, Belgium) high-resolution micro-computed tomography (μCT) scanner, equipped with a 50 kV/40 W X-ray source and a 1.3 megapixel X-ray sensitive CCD camera, was used to assess the 3D microstructure of PCL scaffolds produced at different temperatures and with different filament diameters, in order to evaluate the influence of these production parameters. The 3D microstructure of the multi-material scaffolds was also assessed. PCL scaffolds (Figure 1) with the TMJ disc shape were individually scanned using 180° rotation, with a 0.9° step, around the mediolateral axis, resulting in 210 projection images. The accelerating voltage was 50 kV and the beam current was 800 μA. The exposure time for PCL scaffolds was 4200 and 6000 ms for the multi-material scaffolds; the image pixel size was 26.32 μm and no filter was used. The 3D reconstruction was performed using NRecon (version 1.7.0.4, Bruker), while the morphological analysis was achieved with CT-Analyzer (CTAn, version 1.16.4.1, Bruker). The morphological analysis of the produced scaffolds consisted of (i) identifying and defining a region of interest (ROI) in the 2D projections and (ii) selecting the intensity level (0 to 255) thresholds that correspond to the scaffold material (only) in the projections. Through the morphological analysis, one can obtain several parameters to describe the scaffolds: (i) total volume of interest (VOI), (ii) object volume (OV) and (iii) surface (OS), (iv) number (N_*cp*_), (v) surface (S_*cp*_), (vi) volume (V_*cp*_) of closed pores, and (vii) volume of open pore space (V_*op*_). Thus, it is possible to determine scaffold porosity, which is given by:

(3) P(%)=(VOI-OV)/VOI×100

and the percentage of open porosity, which is given by:

(4) $$\text{OP}\left(\%\right)={\text{V}}_{\text{OP}}/\text{VOI}\times 100$$

### Contact Angle

Contact Angle (CA) assay is a quantitative measure of the wettability of a solid by a liquid. It depends on the surface energy. Higher surface energies are associated with lower CAs. The wettability of the specimens was evaluated by static CA measurement on a Theta Lite optical tensiometer (Attension, Biolin Scientific, Espoo, Finland). A sessile drop methodology was used. A water droplet was poured on the surface of the samples and the CA was measured using a OneAttension 1.0. software (Attension).

### Statistical Analysis

Results are presented as mean ± standard deviation (SD). The statistical analysis was performed using the analytical features of GraphPad Prism 8. Statistically significant differences between independent samples were assessed using a one-way ANOVA with multiple comparisons corrected by the Dunnett test. Replicates of each sample (at least *N* = 3) were performed and statistically different values were considered for *p*-value < 0.05 (*p < 0.05, **p < 0.01, and ***p < 0.001).

## Results and Discussion

**FIGURE 2:**
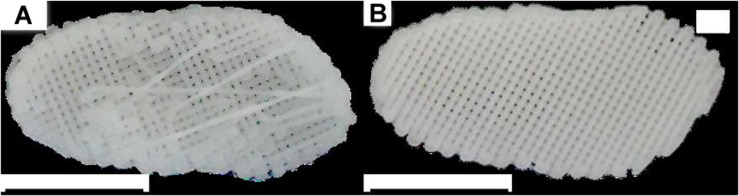
Temporomandibular Joint (TMJ) disc. **(A,B)** views of the produced PCL scaffolds; the scale bar is equivalent to 10 mm.

The proposed PCL scaffolds were successfully produced, presenting a correspondent geometry to the native sheep TMJ disc (Figure 2), as presented in Ângelo et al. (2016). Thickness differences between the bands and the intermediate zone were noticeable, as expected from the analysis of the upper layers of the scaffolds.

**FIGURE 3:**
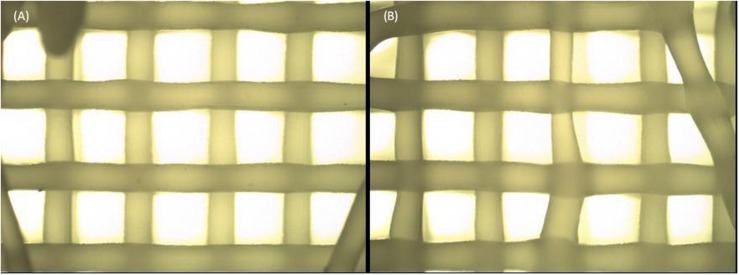
Scaffold morphology. Scaffold with a homogeneous structure **(A)** compared to a scaffold presenting stretched filaments, “S” filaments and exaggeratedly large pores **(B)**.

To avoid widely dispersed and invalid results, only scaffolds within certain structural requirements were selected for the subsequent tests. The selection method was based on a macroscopic and microscopic analysis, and consisted of removing all scaffolds with (i) a flat base (presenting a reduced layer thickness and closed pores), (ii) stretched, curly or “S” filaments, and/or (iii) filament diameters and pore dimensions presenting a deviation greater than 15% from the target (Figure 3).

Poly(ε-caprolactone) scaffolds were successfully produced using all the desired temperatures. However, to properly produce scaffolds for group T78, it was necessary to slow the process, compared to groups T80 and T86, because at this temperature (78°C) the material solidifies faster and tends to follow the movement of the nozzle, generating stretched filaments (Figure 3B). Thus, spindle and crosshead speeds were increased from group T78 (10.5 rpm and 9 mm⋅s^–1^, respectively) to the other groups (14.5 rpm and 12.0 mm⋅s^–1^, respectively). Despite this change, the ratio between these two speeds was maintained.

Scaffolds with different filament diameters were produced using different parameters, namely nozzle diameter, spindle speed and crosshead speed. The spindle speed used to produce the scaffolds of the groups ∅200 and BIO ∅200 was almost five times higher (50 rpm) when compared to ∅300 and BIO ∅300. Since the nozzle diameter is smaller, higher resistance to material flow is created. Thus, the increase in the spindle speed promoted the material flow. On the other hand, crosshead speed was inferior (5 mm⋅s^–1^) to increase the amount of deposited material, to avoid that the material follows the nozzle movement, resulting in stretched filaments.

Since the objective is to obtain scaffolds with similar dimensions, the code for the scaffolds in groups ∅200 and BIO ∅200 was changed to produce 19 layers, while the scaffolds with the other diameter only had 13 layers. However, the scaffolds for the groups with a 200 μm of fiber diameter presented approximately 10% higher thickness than the other groups (3.64 ± 0.07 mm and 3.22 ± 0.10 mm, *p* < 0.05).

It was expected that ∅200 scaffolds would present higher porosity since filament diameter was smaller and the pore area (transverse plane) is larger, compared to the ∅300 scaffolds [(137.8 ± 15.5)⋅10^3^ μm^2^ and (80.3 ± 12.4)⋅10^3^ μm^2^, *p* < 0.05]. However, scaffolds from group ∅200 present higher number of layers throughout the thickness and the pore size in the coronal and sagittal plans probably have a lower area. In the end, both scaffolds have roughly the same volume of material per volume of scaffold ratio, i.e., they present approximately the same porosity (∼63%).

The selected scaffolds were morphologically analyzed. This analysis consisted of determining the anteroposterior and mediolateral dimensions, thickness and base area of the scaffolds, and the average size of the filaments and pores. The scaffolds’ final dimensions were 25.74 ± 0.12 mm and 12.77 ± 0.02 mm in the mediolateral and anteroposterior directions, respectively, and the base area was 298 ± 12 mm^2^.

Using μCT it was also possible to analyze the surface per volume ratio (SVR) and porosity of the scaffolds. SVR was considerably different among the different groups. The group T86 clearly present the lowest SVR (0.83 × 10^–2^ μm^–1^), while the ∅200 group has the highest value (1.68 × 10^–2^ μm^–1^, twice the SVR of the group T86). With exception of the T86 group (∼31.2%), all groups have roughly the same porosity, between 60.1 and 62.8% (twice the porosity of the group T86). These values are relatively close to the TMJ disc reference porosity (70%). Moreover, the reduced or non-existent number of closed pores is indicative of a very high interconnectivity in the produced structures, almost 100%, which allows a good migration of cells throughout the whole structure upon implantation and provides the necessary conditions for a uniform native tissue reconstitution.

Several studies in the literature investigated the influence of different pore sizes in the adhesion, proliferation and migration of different types of cells (Moura et al., 2015). Oh et al. (2007) investigated the effect of pore size on cell and tissue integration, in PCL scaffolds. Three types of cells were used, (i) osteoblasts, (ii) chondrocytes, and (iii) fibroblasts. They reported that the ideal pore size for chondrocytes and osteoblasts growth is in the 300 to 400 μm range, whereas fibroblasts should be approximately 200 μm. However, fibroblasts do not present a significant difference in terms of proliferation in scaffolds with other pore dimensions (100 to 400 μm). Considering the heterogeneity of TMJ disc cellular population (fibroblasts and chondrocytes) (Athanasiou et al., 2009), it can be stated that the ideal pore size, in PCL scaffolds, for TMJ disc TE is between 200 and 400 μm. This supports the pore size used in the present study (268 to 379 μm).

In addition to pore size, it is necessary to consider the surface area, which should have appropriate dimensions for cell adhesion. While chondrocytes and osteoblasts tend to choose larger pores, which provide better conditions for nutrient and metabolite diffusion, fibroblasts prefer lower pore dimensions and greater surface area for cell adhesion and signaling (Oh et al., 2007). This hypothesis is consistent with the fact that the TMJ disc is avascular and approximately 70% of its cell population consists of fibroblasts. Taking this into account, for TMJ disc TE, scaffolds from group T78/∅300 seem to present the adequate pore size (284 ± 31 μm) and SVR (1.53 × 10^–2^ μm^–1^). However, in addition to the morphological characteristics, the mechanical properties must also be considered, and they should be close to the properties of the native TMJ disc.

The mechanical evaluation of the scaffolds shows stress-strain curves with similar behavior across the different groups, characteristic of the adaptation of the native sheep TMJ disc shape to the geometry of the scaffold, in addition to the material used and the mentioned geometric properties. All PCL scaffolds presented a superior compressive modulus compared to the native disc (compressive modulus of 0.1–10 MPa (Athanasiou et al., 2009); yield stress of 1.91 MPa), although the scaffolds with 200 μm filaments present a closer value.

**FIGURE 4:**
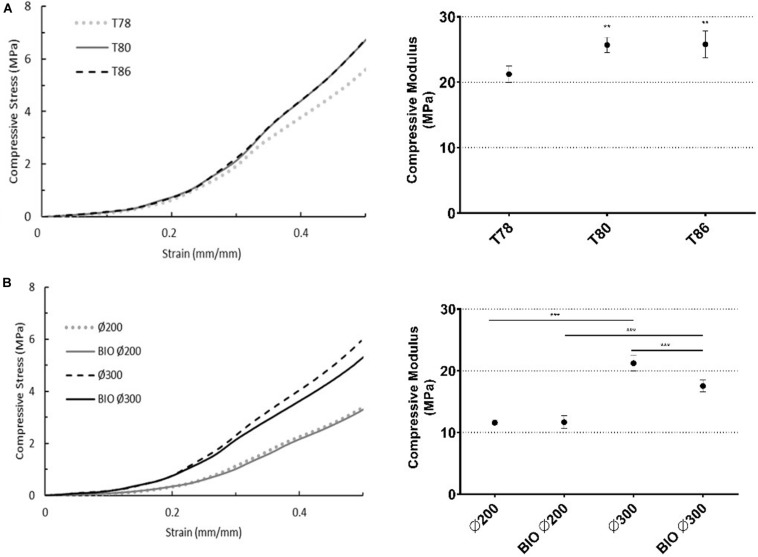
Scaffold compression properties (*n* = 3). **(A)** Influence of nozzle temperature [78–86°C]. **(B)** Influence of filament diameter [∅200 and ∅300] and the surrounding environment (37°C) [BIO ∅200 and BIO ∅300]. Typical stress-strain curve (left) for each sample and respective compression modulus, calculated by the slope of the linear region (right). Statistical differences are presented by **p < 0.01 and ***p < 0.001.

Comparing the scaffolds produced at different temperatures, scaffolds from group T78 showed a 20% lower compressive modulus, 26 MPa (*p* < 0.05, Figure 4A), than the other two groups with the same filament diameter. Moreover, the yield stress increased with temperature (ranging from 2.67 to 3.44 MPa). These results can be explained by the fact that higher nozzle temperatures cause slower solidification, resulting in flat filaments and/or slipping of viscous, non-solidified material. Consequently, structures present a lower thickness and porosity, which leads to an increase in compressive stiffness and strength (Domingos et al., 2012). The decrease in thickness occurs mainly in the central region, where heat transfer to the exterior is slower causing a concavity in the scaffold. Thus, the maximum thickness and the surface filaments are unchanged, so it is only possible to justify these results (morphologically) based on the scaffolds’ porosity. According to the results, the compressive modulus of groups T80 and T86 do not present significant differences between each other (25.68 ± 1.16 and 25.78 ± 2.03 MPa, respectively). However, when compared to T78 (21.22 ± 1.25 MPa) there are significant differences (*p* < 0.01, Figure 4A, right). These results may be due to changes in the material during production with extrusion temperatures above 78°C. Although there were no significant morphological differences between groups T78 and T80, the fact that different spindle and crosshead speeds were used may also have had some impact on the material. Further characterizations using Fourier-transform infrared spectroscopy (FTIR) and thermal analysis after material processing may help to clarify the obtained results.

Despite the high similarity between the two groups with respect to porosity, mechanical assessment showed that the compressive modulus of group ∅300 is twice that of the group ∅200 (11.59 ± 0.36 MPa), *p* < 0.05 (Figure 4B). Moreover, the yield stress decreased by approximately 25% from ∅300 (2.67 ± 0.18 MPa) to ∅200 (1.98 ± 0.16 MPa, *p* < 0.05). This is probably due to the fact that the pores of ∅200 have, in the transversal plane, a pore with an area ∼1.7 times larger than the area of the ∅300 pores and the compressive load is applied perpendicularly to this plane.

The simulation of the hydrated environment led to significant mechanical changes in the compressive modulus of the scaffolds with a 300 μm filament diameter (21.22 ± 1.25 to 17.54 ± 0.95 MPa, *p* < 0.05), whereas for scaffolds with 200 μm filaments, unlike what was expected, there was no significant changes (11.59 ± 0.36 to 11.67 ± 1.04 MPa) (Figure 4B). In both cases, the effect of the surrounding environment was not reflected in significant differences in the yield stress. After 24 h in water at 37°C, scaffolds from group BIO ∅300 presented a compressive modulus almost 20% lower than the scaffolds from group ∅300. For scaffolds with a 300 μm filament diameter, the combination of water and temperature seems to soften the material, reducing its compressive stiffness and, thus, the corresponding compressive modulus. These differences may be associated with the filament diameter, pore size or both and the manner in which heat is transferred and fluid flow occurs in the scaffolds’ structure. For conclusive results, further tests are required to evaluate the effect of the biological environment over time on scaffolds with different filament diameters and pore sizes. Although there are no significant differences when compared to scaffold with a 200 μm filament diameter, there appears to be a marked change in the compressive behavior of these scaffolds under biologic conditions, at least for scaffolds with a filament diameter of 300 μm or greater and/or a pore size less than or equal to 300 μm.

Hydrogels are polymeric networks with viscoelastic behavior, highly hydrophilic and with the capacity to reach high water content, 90–99% (Lee and Kim, 2018). Studies have shown that PEGDA hydrogels have high cellular viability, including for fibroblasts (Liao et al., 2008; Mazzoccoli et al., 2010). Mazzoccoli et al. (2010) tested PEGDA hydrogels with two polymer concentrations, 20 and 40% V/V. Hydrogels with lower concentration presented better viability than those with higher polymer concentration. Nguyen et al. (2012) verified that with the increase of PEGDA MW, the compressive properties decrease. Thus, in this work, a PEGDA hydrogel with a medium MW was used at 20% (V/V). The tested hydrogels presented a compressive modulus of 0.21 ± 0.02 MPa and a maximum compressive stress of 0.20 ± 0.04 MPa. These results are within the range of values found in the literature. The obtained compressive modulus is in the range of reported values for the native TMJ disc (0.1–10 MPa) (Parlato et al., 2014; Nakao et al., 2015).

Since PCL scaffolds presented higher mechanical properties than the native tissue of the TMJ and the PEGDA hydrogel could not withstand the required load, a multi-material approach was tested, joining the best of both worlds. In this way, we hypothesized that a PCL scaffold (∅200) would confer the necessary strength to compression loads and (i) a shell or (ii) a core of PEGDA hydrogel would provide the necessary lubrication and diminish friction.

Poly(ε-caprolactone) has a hydrophobic nature (Moura et al., 2016), so for PEGDA hydrogel to adhere to PCL scaffolds, it was necessary to change this behavior to a hydrophilic one. CA assessment proved its hydrophobic nature (94.4 ± 4.3°). PCL scaffolds were submerged in a NaOH solution for 24 h. After this period, the sessile drops were immediately absorbed upon contact with the scaffold. These results are due to the increase of hydrophilicity promoted by alkaline hydrolysis that contributed to additional carboxylate (−COO-) and hydroxyl (−OH-) groups at the PCL chain termini. Moreover, alkaline hydrolysis induces a superficial erosion, increasing surface roughness and, consequently, the scaffolds’ SVR.

Multi-material scaffolds were morphologically analyzed. The final dimensions of the scaffolds with a (i) PEGDA shell were 27.43 ± 0.76 and 15.30 ± 0.59 mm in the mediolateral and anteroposterior directions, respectively. The base area was 326.28 ± 20.60 mm^2^, and the thickness was 3.91 ± 0.29 mm. The dimensions of the scaffolds with (ii) a core of PEGDA were 26.21 ± 0.17 and 13.37 ± 0.32 mm in the mediolateral and anteroposterior directions, respectively. The base area was 267 ± 4.9 mm^2^ and the thickness was 3.61 ± 0.46 mm.

**FIGURE 5:**
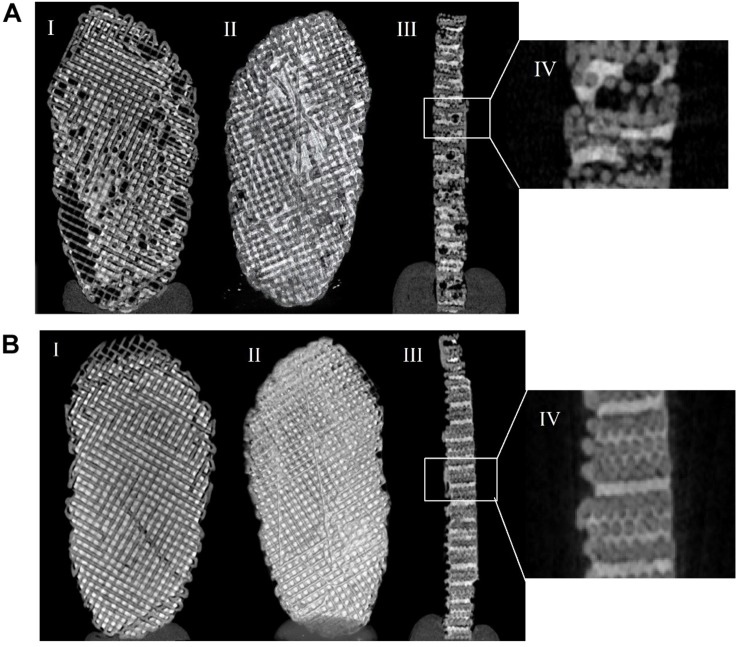
Micro-computed tomography (μCT) analysis of PCL scaffold with a PEGDA shell **(A)** and a PEGDA core **(B)**. Scan of the scaffold (I), scaffold sectioned image (II), cross-section of the scaffold (III), and ampliation of a cross-section of the scaffold (IV).

The porosity of the scaffold with a (i) PEGDA shell was 16.1% and with a (ii) PEGDA core was 1.9%. From analyzing Figure 5, it is possible to confirm the values of porosity obtained since, in the PEGDA core, the hydrogel integrated more evenly in the scaffold pores. In the PEGDA shell, it is more difficult to control the entry of the hydrogel into the pores.

**FIGURE 6:**
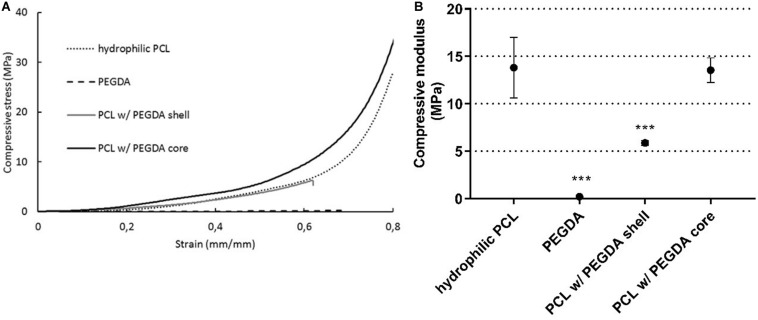
Scaffold properties to compression (*n* = 3): (i) hydrophilic PCL; (ii) PEGDA hydrogel; (iii) Multi-material approach of PCL with PEGDA hydrogel shell; (iv) Multi-material approach of PCL with PEGDA hydrogel core. Typical stress-strain curve **(A)** for each sample and respective compression modulus, calculated by the slope of the linear region **(B)**. Statistical differences are presented by ***p < 0.001.

To fully understand the influence of each parameter individually, mechanical tests were performed on PEGDA hydrogel, ∅200 PCL scaffolds after NaOH treatment (hydrophilic PCL) and on the multi-material scaffolds. The results are summarized in Figure 6.

According to the results obtained, NaOH did not influence the final mechanical properties of the ∅200 scaffolds (13.81 ± 3.20 MPa). Comparing this with the final compressive modulus of the scaffolds with a PEGDA shell (5.87 ± 0.25 MPa), there was a decrease of approximately 57%, while for the scaffolds with PEGDA core there was no significant differences (13.53 ± 1.30 MPa). The multi-material scaffolds with a hydrogel core resulted in a compressive modulus closer to the native disc, leading to improved mechanical properties. As seen in Figure 6, the PEGDA shell-like layer serves as the superficial layers of the TMJ, (i) allowing storage and diffusion of synovial fluid, due to its high water retention capability, (ii) contributing to the reduction of friction, mimicking the HA-lubricin mechanism and (iii) acting as a trampoline, spreading the force and reducing stress concentration on the directly loaded region. On the other hand, the hydrogel as a core works as an internal bumper, meaning that it has the capacity to support a large amount of force during a greater period of time, maintaining the integrity of the whole structure. This is an essential and preferential feature to provide a long-term solution for TMD.

## Conclusion

Tissue Engineering of the TMJ disc is a promising field that can lead to alternatives to the current treatments for TMD. Combining different materials to mimic the properties of the TMJ disc can help restore function due to the lack of capacity for regenerating and self-repairing the TMJ disc.

In this study different parameters were evaluated in the production of the scaffolds, to find a proper implant that mimics the properties of the native disc, regarding its mechanical properties. The results showed that the mechanical properties of the materials can be tailored to better mimic the native properties of the tissues. To sustain the results obtained, dynamic mechanical testing should be performed. Through careful assessment of the different approaches presented, it is possible to conclude that the multi-material strategy of a PCL scaffold with a PEGDA hydrogel is the most promising long-term solution for patients with TMJ disc dysfunctions, despite *in vitro* and *in vivo* testing being required to validate this approach. The proposed scaffold could be the first step toward discovering an effective treatment and a consequent improvement in the quality of life for patients.

## Data Availability Statement

The raw data supporting the conclusions of this article will be made available by the corresponding author, without undue reservation, to any qualified researcher.

## Author Contributions

All authors contributed to the conception and design of the study, manuscript revision, and read and approved the submitted version. CM, DT, and LF collected and organized the data, and wrote sections of the manuscript. CM performed the statistical analysis. DÂ provided the native TMJ discs and wrote part of some sections of the manuscript.

## Conflict of Interest

The authors declare that the research was conducted in the absence of any commercial or financial relationships that could be construed as a potential conflict of interest.
